# Mapping of quantitative trait loci for grain yield and its components in a US popular winter wheat TAM 111 using 90K SNPs

**DOI:** 10.1371/journal.pone.0189669

**Published:** 2017-12-21

**Authors:** Silvano O. Assanga, Maria Fuentealba, Guorong Zhang, ChorTee Tan, Smit Dhakal, Jackie C. Rudd, Amir M. H. Ibrahim, Qingwu Xue, Scott Haley, Jianli Chen, Shiaoman Chao, Jason Baker, Kirk Jessup, Shuyu Liu

**Affiliations:** 1 Texas A&M AgriLife Research, Amarillo, Texas, United States of America; 2 Department of Soil and Crop Science, Texas A&M University, College Station, Texas, United States of America; 3 Agricultural Research Center-Hays, Kansas State University, Hays, Kansas, United States of America; 4 Soil and Crop Sciences Department, Colorado State University, Fort Collins, Colorado, United States of America; 5 Department of Plant, Soil and Entomological Sciences, University of Idaho Aberdeen Research and Extension Center, Aberdeen, Idaho, United States of America; 6 USDAARS Bioscience Research Laboratory, Fargo, North Dakota, United States of America; Institute of Genetics and Developmental Biology Chinese Academy of Sciences, CHINA

## Abstract

Stable quantitative trait loci (QTL) are important for deployment in marker assisted selection in wheat (*Triticum aestivum* L.) and other crops. We reported QTL discovery in wheat using a population of 217 recombinant inbred lines and multiple statistical approach including multi-environment, multi-trait and epistatic interactions analysis. We detected nine consistent QTL linked to different traits on chromosomes 1A, 2A, 2B, 5A, 5B, 6A, 6B and 7A. Grain yield QTL were detected on chromosomes 2B.1 and 5B across three or four models of GenStat, MapQTL, and QTLNetwork while the QTL on chromosomes 5A.1, 6A.2, and 7A.1 were only significant with yield from one or two models. The phenotypic variation explained (PVE) by the QTL on 2B.1 ranged from 3.3–25.1% based on single and multi-environment models in GenStat and was pleiotropic or co-located with maturity (days to heading) and yield related traits (test weight, thousand kernel weight, harvest index). The QTL on 5B at 211 cM had PVE range of 1.8–9.3% and had no significant pleiotropic effects. Other consistent QTL detected in this study were linked to yield related traits and agronomic traits. The QTL on 1A was consistent for the number of spikes m^-2^ across environments and all the four analysis models with a PVE range of 5.8–8.6%. QTL for kernels spike^-1^ were found in chromosomes 1A, 2A.1, 2B.1, 6A.2, and 7A.1 with PVE ranged from 5.6–12.8% while QTL for thousand kernel weight were located on chromosomes 1A, 2B.1, 5A.1, 6A.2, 6B.1 and 7A.1 with PVEranged from 2.7–19.5%. Among the consistent QTL, five QTL had significant epistatic interactions (additive × additive) at least for one trait and none revealed significant additive × additive × environment interactions. Comparative analysis revealed that the region within the confidence interval of the QTL on 5B from 211.4–244.2 cM is also linked to genes for aspartate-semialdehyde dehydrogenase, splicing regulatory glutamine/lysine-rich protein 1 isoform X1, and UDP-glucose 6-dehydrogenase 1-like isoform X1. The stable QTL could be important for further validation, high throughput SNP development, and marker-assisted selection (MAS) in wheat.

## Introduction

Identification of consistent loci or haplotypes associated with traits is important in generation advancement decision, forward breeding, gene pyramiding and deployment recommendations in wheat and other crop breedings. These genomic regions can also play an important role during placement of new wheat variety into specific geographical footprints to maximize yield performance via genotype-environment matching. Several factors are considered prior to deployment of a QTL including the stability of the QTL across environment and genetic background, equivalency or effect of the QTL on other traits in elite germplasm, efficacy of the QTL if it is linked to disease or pest resistance, and availability of diagnostic marker in linkage disequilibrium with the QTL. In wheat, several strategies for MAS have been used to improve traits with majority focusing on simple traits [1, 2]. Notably, *Rht-B1* and *Rht-D1* linked to reduced stature have been deployed via MAS in wheat and in some environments a concomitant increase in yield have been observed although there seem to be a strong linkage disequilibrium between the two dwarfing genes and Fusarium head blight (FHB) susceptibility [2, 3]. A recently mapped QTL for reduced height, *Rht24*, on chromosome 6A has a potential for MAS and a diagnostic KASP marker has been developed [2, 4]. Our previous work using RIL population and 90K SNP array detected stable diagnostic marker tightly linked to *Wsm2*, a gene conferring resistance to wheat streak mosaic virus and is currently applied in MAS in winter wheat [5, 6]. Markers linked to *Fhb1 and Qfhs*.*ifa5A*, conferring resistance to Fusarium head blight, have also been applied in MAS in wheat breeding [7]. In addition, several other functional markers have been developed for agronomic traits, end-use quality and disease resistance [7, 8]

Detection of consistent QTL depends, in part, on the quality of phenotypic data, genotypic data, and statistical analysis. Typically, evaluation of mapping population is done over multiple seasons in different testing footprints that are representatives of target mega-environments. This approach facilitates detection of consistent QTL over environments and seasons by modeling multi-environment variance-covariance structures that account for heterogeneity of genetic variance. Recent advances in marker technology have led to development of dense and ultra-dense genetic maps providing a fairly good genome coverage [9]. In addition, advances in statistical modeling particularly application of linear mixed models (LMM) provides flexibility to include variance-covariance (VCOV) structure to account for heterogeneity in genetic variances and environmental correlation [10, 11]. LMM can account for both QTL-by-trait interactions (QTI) and QTL-by-environment interactions (QEI) including interaction with environmental covariables such as temperature, light duration and intensity, and moisture levels [10, 12].

Many genetic studies focusing on QTL discovery in wheat have utilized sparse genetic maps to tag QTL and rely on specific statistical method limiting the interpretation of the results to assumptions defined by the analysis approach. In addition, most studies assume the significance of epistatic and pleiotropic interactions underlying yield and yield components in wheat. Most of these QTL have been summarized and posted online on the catalogue of gene symbols. (https://shigen.nig.ac.jp/wheat/komugi/genes/symbolClassList.jsp). In the present study, we used high resolution genetic maps constructed using 90K array SNPs and implemented linear and linear mixed model QTL mapping approach in different genetic mapping software programs [13–16]. The SNP associated with significant QTL were used in combined analysis of pleiotropy and epistasis as outlined by Tyler et al. [13]. Our objectives were to map stable QTL for grain yield and yield components in one of the most popular hard red winter wheat, TAM 111, within the framework of single trait multi-environment and multi-trait analysis and examine marker-to-trait effects and marker-to-marker interactions through analysis of pleiotropy and epistasis [13, 16]. Consistent QTL detected from various statistical models were compared for their locations on consensus genetic and physical maps.

## Materials and methods

### Recombinant inbred line population and trial evaluation

A bi-parental mapping population was derived from a cross between CO960293-2 (PI 615160) [17] and TAM 111 (PI 631352) [18]. CO960293-2 was developed by Colorado Agricultural Experiment Station and co-released as a germplasm line by Colorado and Kansas Agricultural Experiment Stations primarily for resistance to wheat streak mosaic virus and Russian Wheat Aphid (*Diuraphis noxi* M.). TAM 111 is a popular cultivar developed and released by Texas A&M AgriLife Research in 2002. It has good performance in both low and high productivity environments [18, 19]. A trial comprising 217 recombinant inbred lines (RIL) plus three checks (four checks in 2012/13) was planted in eight environments from 2012 to 2014; each location-by-year combination was considered as an environment. The locations used in this study were in Texas at Texas AgriLife Research stations in Bushland (35° 06' N, 102° 27' W), Chillicothe (34° 07' N, 99° 18' W) and Etter (35° 59' N, 101° 59' W); Kansas State University Agricultural Research Center-Hays, Hays KS (38° 51' N, 99°20' W); University of Idaho Aberdeen Research and Extension Center, Aberdeen ID (42° 57' N, 112° 49' W); and Colorado State University Plainsman Research Center, Walsh CO (37° 25' N, 102° 18' W). The trials in Etter and Hays were evaluated in both the 2012/13 and 2013/14 crop seasons. The trials in Etter, Aberdeen and Walsh were under well-watered conditions whereas the remaining trials were under dryland conditions. The plot size was 4.645 m^2^ (4.459 m^2^ in Walsh) and all trials had two replications. Standard agronomic practices were carried out for each environment.

### Trait measurements and data analyses

Grain yield was recorded in all the environments whereas yield components, plant height and days to heading were recorded in a subset of environments. Yield components were recorded in the Texas environments and Hays 2013 (HY13). Plant height was recorded in all the environments except Walsh 2014 (WA14) and Etter 2013 (ET13) whereas days to heading was recorded in ET13, HY13, Aberdeen 2013 (AB13) and Bushland 2014 (BS14). Days to heading was recorded at Feekes growth stage 10.1 as the number of days from January 1^st^ to when 50% of the spikes had emerged from the boot. Percentage of green leaf area was visually rated in BS14 and Chillicothe 2014 (CH14) at Feekes growth stage 10.5 on a scale of 0–100%, where 0% = all the leaves senesced and 100% = all leaves green. Similarly, the greenness of the flag leaf was rated at Feekes growth stage 10.5 on a scale of 0–100% where 0% = whole flag leaf senesced and 100% = whole flag leaf green. At Feekes growth stage 11, plant height was measured in centimeters (cm) from representative plants in each plot as the distance from the base of the stem to the tip of the spike excluding awns. In addition, a half meter long sample from a uniformly filled and representative inner row was harvested from each plot and used for determination of biomass and yield components. The samples were oven-dried at 60°C for three days and the weight of each sample recorded. The total number of stems and the number of heads were counted for each biomass sample. The spikes m^2^, mean single head weight, kernels spike^1^, kernels m^2^, and thousand kernel weight were calculated from the plot sample. The spikes m^2^ was computed by dividing the number of heads by the sample plot area. The thousand kernel weight was determined by counting and weighing three sets of 100 kernels for each plot and multiplying the average weight by 10. Mean single head weight was calculated by dividing the total dry head weight per plot biomass sample by the number of heads. The kernels m^-2^ was estimated by dividing the weight of grain from the sample by single kernel weight. The harvest index was calculated as grain weight per sample divided by total weight of biomass. All trials were harvested using a combine harvester and the grain yield plot^-1^ was used to calculate yield in metric tons hectare^-1^. Test weight, in kg m^-3^, was measured using Seedburo equipment (www.seedburo.com, Des Plaines, IL, USA).

Individual and combined environment data was subjected to analysis of variance (ANOVA) in SAS (SAS Institute, 2013) to determine the significance of genotypic and other components of the model. The statistical model used for individual environment analysis was as follows:

$$Y_{ik}=\mu +R_{k}+G_{i}+\varepsilon_{ik}$$

Where *Y*_*ik*_ is the observed phenotypic value of the *i*^*th*^ genotype in *k*^*th*^ replicate, μ is the overall mean, *R*_*k*_ is the replication effect, *G*_*i*_ is the genetic effect of *i*^*th*^ genotype and Ɛ_ik_ is the residual. All components were considered fixed. The statistical model for combined analysis of variance was as follows:

$$Y_{ijk}=\mu +R\left(E\right)+G_{i}+E_{j}+{\left(GEI\right)}_{ij}+\varepsilon_{ijk}$$

Where *Y*_*ijk*_ is the observed value of the *i*^*th*^ genotype in the *j*^*th*^ environment and *k*^*th*^ replicate, *R(E)* is replication nested within the environment, *E*_*j*_ is the effect of the environment, *(GEI)*_*ij*_ is genotype-by-environment interaction, Ɛ_ijk_ is the residual. To compute mean squares, all the components were considered as fixed whereas for variance components, all components in the model were considered as random. Best linear unbiased predictors (BLUP) and variance components were computed using residual maximum likelihood adapted to META-R program [20]. The BLUP were used for QTL analysis in GenStat version 17 [15]. For single trait multi-environment QTL analysis, the appropriate VCOV structure was modeled in GenStat to account for heterogeneity of genetic variances and correlation among environments [10, 12]. The best VCOV was selected based on the Schwarz information criterion. The genetic correlations (ρ_g_) between pairs of traits were computed using the following formula in METAR:

$$\rho_{g}=\frac{{\mathrm{Cov}}_{x,y}}{{\left(\sigma_{x}^{2}\sigma_{y}^{2}\right)}^{1/2}}$$

Where *COV*_*x*,*y*_ is the genetic covariance between trait *x* and *y*, *σ*^*2*^_*x*_ is the variance of phenotype *x* and *σ*^*2*^_*y*_ is the variance of phenotype *y* [20]. Entry-mean heritability estimates were computed using the formula:

Combinedenvironmenth2=σg2σe2rt+σge2t+σg2

Where *r* is the number of replication, *t* is the number of environments, *σ*^*2*^_*g*_ is genotype variance, *σ*^*2*^_*ge*_ is the GEI variance, and *σ*^*2*^_*e*_ is the residual variance [21]

### DNA extraction and genotyping

Total genomic DNA was extracted from leaf samples of 217 RIL using a CTAB method with minor modification [22, 23]. The RIL and four sets of each parent were genotyped using 90K SNP array based on the manufacturer’s protocol (www.illumina.com). The fluorescence signal was captured using an Illumina scanner and subsequently processed using GenomeStudio software (www.illumina.com). To adjust clusters with skewed cluster separation, manual curation of the data was done by examining the clusters in a Cartesian plot. Loci with low average normalized intensity and undefined clusters were excluded prior to downstream analysis. The genotype data set consisting of 8,819 high quality SNP was used for downstream statistical analysis [5, 24].

### Linkage mapping and QTL analysis

We implemented linkage mapping in JoinMap version 4.0 [25]. Prior to linkage map construction, SNPs with identical loci scoring at 100% similarity were omitted to eliminate genetic redundancy and improve computation efficiency. In addition, all SNP with significant segregation distortion based on Chi square test (*P* < 0.05) were also omitted. We grouped loci into linkage groups based on Independence LOD with increasing stringency from 2.0 to 30.0 and the incremental step of 1.0. The Kosambi mapping function was used to convert recombination frequency into centiMorgans [26]. The pairwise recombination frequency was calculated based on a maximum likelihood (ML) algorithm with the default settings in JoinMap [25]. The final linkage map of 5,580 SNPs covering all of 21 chromosomes with 44 linkage groups was used for QTL analysis. Linkage groups were assigned to chromosomes based on loci information in the 9K and 40K genetic maps [27, 28].

Multi-environment and multi-trait QTL analyses were performed in GenStat based on a LMM framework as described by several authors [10, 12, 29]. In GenStat, QTL mapping was implemented in a stepwise manner commencing with simple interval mapping followed by at least two rounds of composite interval mapping using QTL identified to control the genetic background [30–32]. Backward selection was conducted on QTL detected and the final effects were estimated based on multi-QTL model [3032]. The markers associated with QTL were used for combined analysis of pleiotropy and epistasis using the CAPE package in R [13, 33, 34].

In addition, MapQTL 6.0 [25] was used to analyze and identify significant QTL for each trait based on single environment data. We used QTLNetwork sofware [16] to analyze the significant QTL additive effects, epistasis effects and their interactions with environments across data from multiple environments. The detected QTL results were compared within the three models (single-environment, multi-environment, and multi-trait) of GenStat. Comparative analyses were conducted based on the linked SNPs of unique and consistent QTL using databases from T3 (https://t3sandbox.org/t3/sandbox/wheat/, accessed on March 1, 2017) and the Chinese Spring reference genome from International Wheat Genome Sequence Consortium (IWGSC RefSeqv1.0, https://wheat-urgi.versailles.inra.fr/Seq-Repository/Assemblies, accessed on March 1, 2017).

## Results

### Phenotypic analysis of yield and its related traits

**Fig 1:**
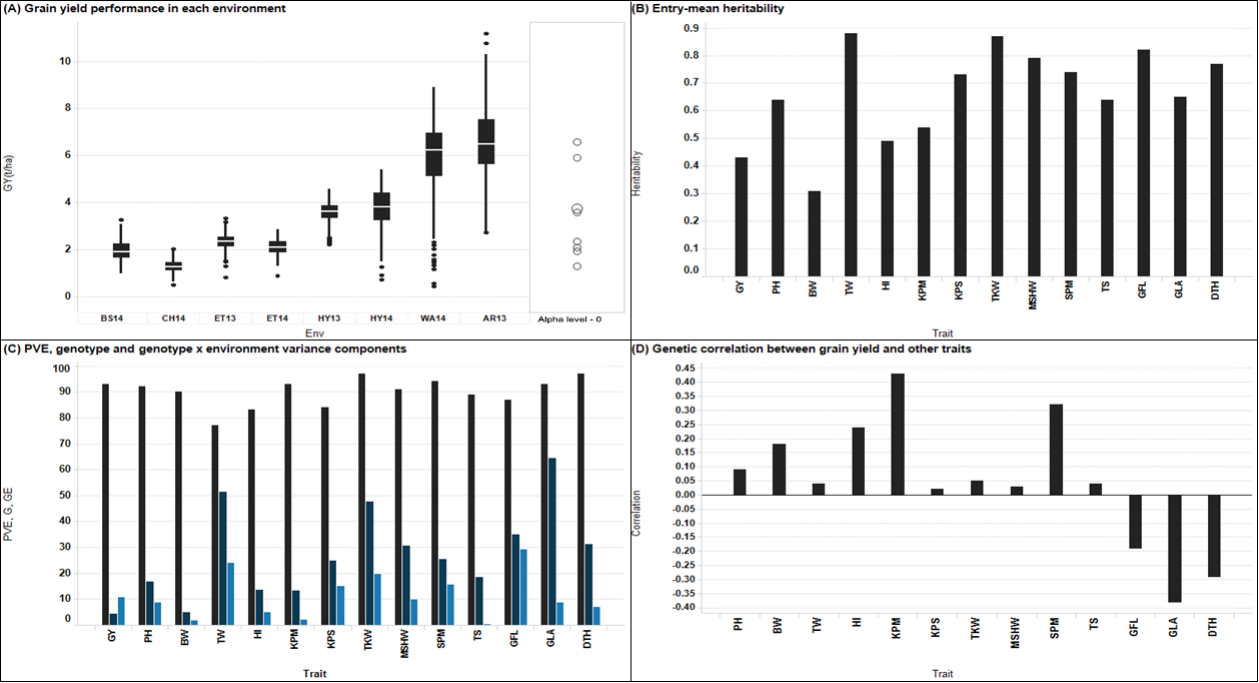
Visualization of phenotypic performance (A) Individual environment boxplot for grain yield. The *y*-axis is grain yield in t ha^1^ and the *x*-axis represents environments. The mid line in the box represent the median, the lower and upper horizontal lines of the box represent 25 and 75 percentiles, respectively. The lower whisker represents the 25^th^ percentile minus 1.5 **×** inter-quartile range (IQR) and the upper whisker is the 75^th^ percentile plus 1.5 **×** IQR. (B) Entry-mean heritability (C) Percentage of phenotypic variance explained (PVE), genotype and genotype x environment components (D) Genetic correlation between grain yield and agronomic traits.

Combined ANOVA across environments revealed significant (*P* < 0.001) genotype and environment components for traits whereas the GEI was statistically significant for all traits except for biomass weight (S1 Table). The genotype × environment variance component for grain yield was higher than variance due to genotype. A reverse trend was observed for all the other traits where genotype variance components were greater than genotype × environment variance (Fig 1C). The highest variance component due to genotype were observed for green leaf area, thousand kernel weight, and test weight. The phenotypic variance explained (PVE) by the model fit was greater than 80% for all the traits except test weight which had PVE of 77% (Fig 1C). The entry-mean heritability ranged from moderate (0.40 to 0.60) to high (> 0.60) except for biomass weight which had heritability of 0.31 (Fig 1B). Moderate estimates of heritability (0.43–0.64) were observed for grain yield, harvest index, and total stems whereas the remaining traits had high heritability (0.65–0.88). The average grain yield across environments was 3.6 t ha^-1^ with a corresponding test weight of 750.4 kg m^-3^. On average, the population had 142 days to heading and the mean plant height was 63.2 cm. The average number of spikes m^-2^ was 376^-^. A single spike had an average of 28 kernels and weighed 0.7 g. The average thousand kernel weight was 26.2 g (S1 Table). We observed high yield variability in performance both within and across environments (Fig 1A). The highest range in yield was observed in AB13 and WA14 environments compared to dryland experiments. Dryland experiments showed differences in phenotypic expression with the trial in CH14 showing the least range in yield performance (0.5 to 2.0 t ha^-1^) attributed to a severe drought stress leading to a narrow window for the grain filling stage. Nonetheless, we still detected significant genotypic variation and the entry-mean heritability was 0.65 (Fig 1A and S1 Table). Comparison of average grain yield showed that environment AR13, WA14, and CH14 were significantly different (P < 0.01) from each other and from all other environments in the present study as indicated by nonoverlapping comparison circles (Fig 1A). BS14 and CH14 were not significant from each other. Similarly, the average grain yield in HY13 and HY14 was not significant from each other. Grain yield under drought stress condition ranged from 1.3 t ha^-1^ in CH14 to 3.9 t ha^-1^ in HY13 (Fig 1A).

Across environments, grain yield had significant genetic correlation (*P* < 0.01) with days to heading, harvest index, kernels m^-2^, biomass weight, spikes m^-2^, green leaf area, and greenness of the flag leaf (Fig 1D). The negative correlation observed for grain yield with both green leaf area and greenness of the flag leaf suggest that delayed chlorophyll decay may not necessarily translate to increased grain yield in the US High Plains. The significant correlation between grain yield and harvest index, biomass weight and spikes m^-2^ suggest that improvement in grain yield may be achievable through indirect selection of these traits. The highest significant correlation across environment (ρ_g_ = 0.43, *P* < 0.01) was observed between grain yield and spikes m^-2^ (Fig 1D). The genetic correlation between grain yield with biomass weight was moderate (ρ_g_ = 0.40 to 0.60) to high (ρ_g_ > 0.60) in all trials where the yield components were recorded (data not shown). Kernels m^-2^ was significantly correlated with grain yield in three environments but was not significantly correlated with grain yield in CH14 (data not shown). Test weight was positively correlated with grain yield in four environments although the correlations were less than 0.30 (data not shown).

### Consistent QTL for grain yield, yield components and agronomic traits

We detected nine consistent QTL on chromosomes 1A, 2A, 2B, 5A, 5B, 6A, 6B, and 7A (Table 1, S2–S6 Tables). The QTL on chromosome 1A was linked to spikes m^-2^ across all the four analysis methods, henceforth referred to as M1, M2, M3 and M4 for method 1 (GenStat-multiEnv), method 2 (GenStat-SingleEnv), method 3 (MapQTL), and method 4 (QTLNetwork), respectively. The magnitude of additive effects and PVE varied from one method to the next. The PVE for spikes m^-2^ in M1 ranged from 5.8–7.9% with an additive effect range of 13.1–19.1 spikes. The PVE for spikes m^-2^ in M2 was 7.8% whereas the range in M3 (6.1–8.1%) was similar to M1. This same QTL was also linked to harvest index in M1 and M3; mean single head weight in M1 and M2; kernels spike^-1^ in M2, M3, and M4; thousand kernel weight in M3 and M4 and test weight in M2 (Table 1).

**Table 1:** Summary of consistent QTL for yield, yield components and agronomic traits.

Chrom	QTL ID	Peak (cM)		M1^b^		M2 ^b^		M3 ^b^		M4 ^b^	Favorable alleles ^e^
			Trait^a^	A ^c^	PVE ^d^	A ^c^	PVE ^d^	A ^c^	PVE ^d^	A ^c^	
1A	1	170–179	KPS			0.82	7.9	0.5–0.8	5.6–7.5	0.6	P2
		173–186	SPM	13.1–19.1	5.8–7.9	14.0	7.8	11.8–18.7	6.1–8.6	9.8	P1
		179–181	TKW					0.6–0.7	9.0	0.3	P2
		201–204	HI	0.006–0.008	3.0–6.1			0.01	8.8		P2
		173–179	MSHW	0.02	4.1–10.9	0.03	7.0–11.5				P2
		196	TW			4.4	5.3				P2
2A.1	3	46–68	KPS	0.83	5.1–8.1	0.8–1.3	8.1–12.2	0.8–1.5	5.6–12.8	0.8	P2
		46–52	TW	6.7–22.0	2.5–12.2	19.6	9.7				P1
2B.1.2	4	122–125	SPM			16.9	9.7	16.7	8.5	7.8	P1
		142	PH	0.5–0.9	2.2–7.4						P2
		142–152	DTH			0.4–0.7	8.3–21				P1
2B.1	5	170–173	GY	0.05–0.25	3.3–25.1	0.12	13.4	0.11	8.6–20.7		P2
		167	KPS					0.7	5.3		P2
		165	SPM	6.8–13.9	1.8–6.0						P1
		166–173	TKW	0.4–1.0	2.7–19.5	0.7	8.6	0.5–1.0	6.1–18.4		P2
		170–173	HI	0.004–0.016	3.3–22.9	0.01–0.02	5.6–7.0	0.01–0.02	9.8–25.9	0.013	P2
		164–168	TW	5.5–8.6	1.8–14.4	6.6	5.9				P2
		167	DTH	0.3–0.9	2.5–30.6						P1
		168–173	GFL	0.1–0.2	10.6–36.4	0.01	21.2–39.5				P1
		168–175	GLA	0.2–0.3	32.0–42.1	0.2	21.1–25.4				P1
5A.1	8	99	GY	0.04–0.25	2.2–5.2						P2
		99	SPM							9.0	P1
		86–117	TKW					0.5–0.8	6.1–8.6	0.52	P2
		100	KPM			126.1	9.0				P1
5B	11	211–243	GY	0.04–0.20	1.8–9.3	0.10	7.2	0.11	7.7–8.2	0.07	P2
6A.2	14	131	GY					0.06	5.2		P2
		129	KPS							0.3	P2
		125–127	SPM			19.5	9.3	20.0	8.8	8.7	P2
		129–133	TKW	0.8–1.1	11.8–17.5	0.7–1.0	12.8–14.0	0.7–1.0	9.3–13.4	0.7	P1
		115–122	HI	0.006	1.3–6.5			0.01	7.1–8.3	0.01	P1
		129	TW			7.3	7.2				P1
		113	TS			2.7	7.2				P2
		130–131	PH	0.8	0.8–6.3	1.1	7.8				P1
		107	GLA	0.1	4						P2
6B.1	15	130–135	SPM	5.5–22.7	1.7–9.4	18.6	8.5	12.9–14.9	6.8–9.5		P1
		133–166	TKW	0.4–0.6	2.5–4.5					0.3	P2
		127–151	TW	4.5–13.5	3.5–5.9	6.7	6.2				P1
		146–148	PH			1.9–2.4	7.7–9.2				P2
7A.1	16	20	GY			0.1	7.4	0.11	7.2		P1
		4–20	KPS	0.5–1.0	2.4–7.3	0.9	8.4	0.5–1.1	5.3–8.6	0.5	P2
		0–20	TKW	0.3	0.9–2.1	0.7	9.5	0.6–0.7	6.2–8.7	0.6	P1

^a^Abbreviation of traits: GY grain yield, TW test weight, DTH days to heading, PH plant height, HI harvest index, SPM spike m^2^, KPS kernels spike^1^, KPM, kernels m^2^, MSHW mean single head weight, TKW, thousand kernel weight, TS total stems, GLA green leaf area, GFL greenness of flag leaf

^b^ M1 Method 1, GenStat Multi-env model; M2 Method 2, GenStat Single-env model; M3 Method 3, MapQTL; M4 Method 4, QTLNetwork

^c^ A Additive effect; Ignore the negative signs because the parental favorable alleles are labelled

^d^ PVE Phenotypic variations explained

^e^ P1 female parent, CO960293-2; P2 male parent, TAM 111

The interval region for this 1A QTL harbors seven protein coding gene with transcript ID *TRIAE_CS42_1AL_TGACv1_001932_AA0036960*, *Traes_1AS_656CB2399*, *Traes_1AS_7B084FDFA*, *Traes_1AS_3160922E9*, *Traes_1AS_6CB929C18*, *Traes_1AS_1CBC21AE8*, *Traes_1AS_5317928F1*. This transcript indicates that the 1A QTL is around the centromere given some genes are on the short arm and others on the long arm (http://plants.ensembl.org).

*The QTL* on chromosome 2A.1 was consistently linked to the number of kernels spike^-1^ with PVE of 5.1–8.1% in M1, 8.1% and 12.2% in M2, and 5.6–12.8% in M3. In all the QTL analysis approach, this 2A.1 QTL had an additive effect of about one kernel per spike. In addition to kernel spike^-1^, it was linked to test weight in M1 and M2 where it had PVE of 2.5–12.2% and 9.7% in M1 and M2, respectively. Comparative search showed that the gene, *Traes_2AL_2EC344DEE*, is within the region of *2A*.*1* QTL on the long arm of chromosome 2A (http://plants.ensembl.org).

Two QTL, repeatable across environment and statistical analysis methods were detected on chromosome 2B.1. The first one was at the region of 122 to 152 cM of *2B*.*1*, was significantly associated with spikes m^-2^ in M2 and M3 with a corresponding PVE of 9.7% and 8.5%, respectively. The additive effect for this QTL was 16 spikes m^-2^ in M2 and M3 and 7.8 spikes m^-2^ in M4. Analysis using M2 showed that this 2B.1 QTL was also linked to day to heading with PVE range of 8.3–20.7%. BLAST search showed that 20 genes falls within the confidence interval of this first QTL on 2B.1 (http://plants.ensembl.org; https://urgi.versailles.inra.fr). The second QTL on chromosome 2B.1 was linked to harvest index in all the four methods but to yield and thousand kernel weight in the first three methods. We also detected significant statistical association of this QTL on test weight, greenness of the flag leaf and green leaf area in both M1 and M2. For grain yield, this 2^nd^ QTL on 2B.1 had a PVE range of 3.3–25.1% in M1, 13.4% in M2, and 8.6–20.7% in M3. Overall, the additive effect for grain yield ranged from 0.05–0.25 t ha^-1^ depending on the environment and statistical analysis method. The PVE for this 2^nd^ QTL on 2B.1 on harvest index was 3.3–22.9% in M1, 5.6% and 7.0% in M2, and 9.8–25.9% in M3. The corresponding additive effect for harvest index was 0.004–0.016 in M1, 0.01 and 0.02 in M2, and 0.01–0.02 in M3. This 2^nd^ QTL on 2B.1 was also linked to days to heading in M1 with a PVE range of 2.5–30.6%. Based on the database search of the linked SNP markers, this QTL is very close to the *PpdB*_*1*_ gene, 1.9 Mb apart on the Chinese Spring reference genome (www.wheatgenome.org) and is associated with protein coding gene heat shock 70kDa protein *TRIAE_CS42_2BS_TGACv1_146035_AA0453700*, *Traes_2BS_96D64756B*, *Traes_2BS_F3D1922E5* and *Traes_2BS_B88CDE912* on the short arm of chromosome 2B (S8 Table) (http://plants.ensembl.org).

The QTL on 5A.1 was associated with yield based on M1 analyses, spikes m^-2^ from M4, and thousand kernel weight from M3 and M4 (Table 1). However, this is a QTL with small effect based on its additive effect and PVE. The peak SNP for *5A*.*1* is within *TRIAE_CS42_5AL_TGACv1_375092_AA1215930* gene which has 7 exons and 20 variants (http://plants.ensembl.org).

The QTL on 5B at 211 cM was consistently linked to grain yield across environments and methods with PVE of 1.8–9.3%, 7.2%, and 7.7–8.2% in M1, M2, and M3, respectively. This QTL had an additive effect of 0.04–0.20 t ha^-1^ in M1, 0.1 t ha^-1^ in M2, 0.11 in M3, and 0.07 in M4. The region spanning *5B*.*1* on the long arm of chromosome 5B harbors *TRIAE_CS42_5BL_TGACv1_408832_AA1364560* gene which has a single transcript coding of 146 amino acid. The QTL on 6A.2 was consistently linked to thousand kernel weight with a corresponding PVE of 11.8–17.5% in M1, 12.8–14.0% in M2, and 9.3–13.4% in M3. In addition, 6A.2 QTL was also linked to harvest index in M1, M3, M4; spikes m^2^ in M2, M3, M4; and plant height in M1 and M2. One of the peak SNP (IWB8924) for *6A*.*2* is within *TRIAE_CS42_6AL_TGACv1_472318_AA1520780* gene on the long arm of chromosome 6A (http://plants.ensembl.org). The gene has four transcripts and codes Diacylglycerol kinase which play a role in signaling under biotic and abiotic stress [35]. Another peak marker, IWB67907, is within a predicted *TRIAE_CS42_6AL_TGACv1_472781_AA1525920* gene that codes Alphamannosidase. In other plants, alphamannosidase has enzymatic function on Nglycans [36, 37]. IWB7004, also within confidence interval for *6A*.*2*, is linked to *TRIAE_CS42_1BS_TGACv1_051115_AA0177850* gene for uncharacterized protein in wheat (http://plants.ensembl.org). The QTL on 6B.1 was significantly associated with spikes m^2^ and had PVE of 1.7–9.4% in M1, 8.5% in M2, and 6.8–9.5% in M3. The additive effect of 6B.1 QTL for spikes m^2^ was 5.5–22.7, 18.6, and 12.9–14.9 spikes m^2^ in M1, M2, and M3, respectively. A search for genes within the QTL region revealed two protein coding genes *TRIAE_CS42_6BL_TGACv1_501196_AA1614830* on 6BL, and *TRIAE_CS42_6BS_TGACv1_514653_AA1662770* on 6BS indicating that the QTL is located near the centromeric region. On chromosome 7A.1, we detected a QTL consistently linked to the number of kernels spike^1^ and thousand kernel weight across environment and QTL analysis methods. The PVE for thousand kernel weight was 0.9–2.1%, 9.5%, and 6.2–8.7% in M1, M2, and M3, respectively. The corresponding additive effect was 0.3 g, 0.7 g, 0.6–0.7 g, respectively. For the number of kernels spike^1^, the PVE was 2.4–7.3%, 8.4%, and 5.3–8.6% in M1, M2, and M3, respectively. BLAST search of peak SNP showed that *TRIAE_CS42_7AL_TGACv1_557259_AA1778880* gene is located within the region spanning *7A*.*1*.

QTLNetwork analyses for additive affects, epistasis, and their interactions revealed more significant effects (Table 1, S2 and S3 Tables). Epistasis was important for thousand kernel weight with seven significant additive × additive (A × A) interaction detected between QTL on chromosome 1A, 1B.1, 3A.3, 3B.1, 5B, 6B and QTL on 5D.1, 7A.3, and 7B. Three out of these seven were between *Qtkw*.*tamu*.*7B*.*2* and a QTL on 1A, 3B.1, and 6B (S3 Table). There was no significant additive × additive × environment (A × A × E) interaction for thousand kernel weight but additive × environment (A × E) interaction was important. Among the ten QTL linked to thousand kernel weight, *Qtkw*.*tamu*.*6A*.*2* showed significant A × E interaction at CH14 (S2 Table). The number of spikes m^-2^ had nine significant epistases detected with one showing significant A × A × E interaction. Five significant A × A interaction for spikes m^-2^ were between one major QTL and a new QTL that was not detected based method 1 to 4 while one epistasis was between two new QTL. We detected one significant epistasis for kernel spike^1^ and harvest index. The latter involved interaction between two new QTL while the former involved interaction between *Qkps*.*tamu*.*3D*.*1* and a new QTL for kernel spike^-1^. Only the A × A × E for two major stable QTL *Qhi*.*tamu*.*2B*.*1* and *Qhi*.*tamu*.*6A*.*2* was significant (S3 Table).

The QTL on 1A, 1B.1, 1B.3, and 2B.1 at 170 cM as well as the QTL on 5B at 243 cM showed significant A × E for grain yield whereas two QTL on 1A and 2B.1 at 170 cM showed significant A × E for harvest index (S2 Table). An interaction between two QTL, *Qgy*.*tamu*.*3D*.*1* and *Qgy*.*tamu*.*5B*.*1*, was not significant for A × A epistasis but significant for A × A × E interactions. Overall, the environment AB13 (Aberdeen, Idaho 2013) was different from other environments. Five out of the six significant A × A × E interactions involved AB13 (S3 Table).

### Multi-trait QTL for yield and yield components

**Fig 2:**
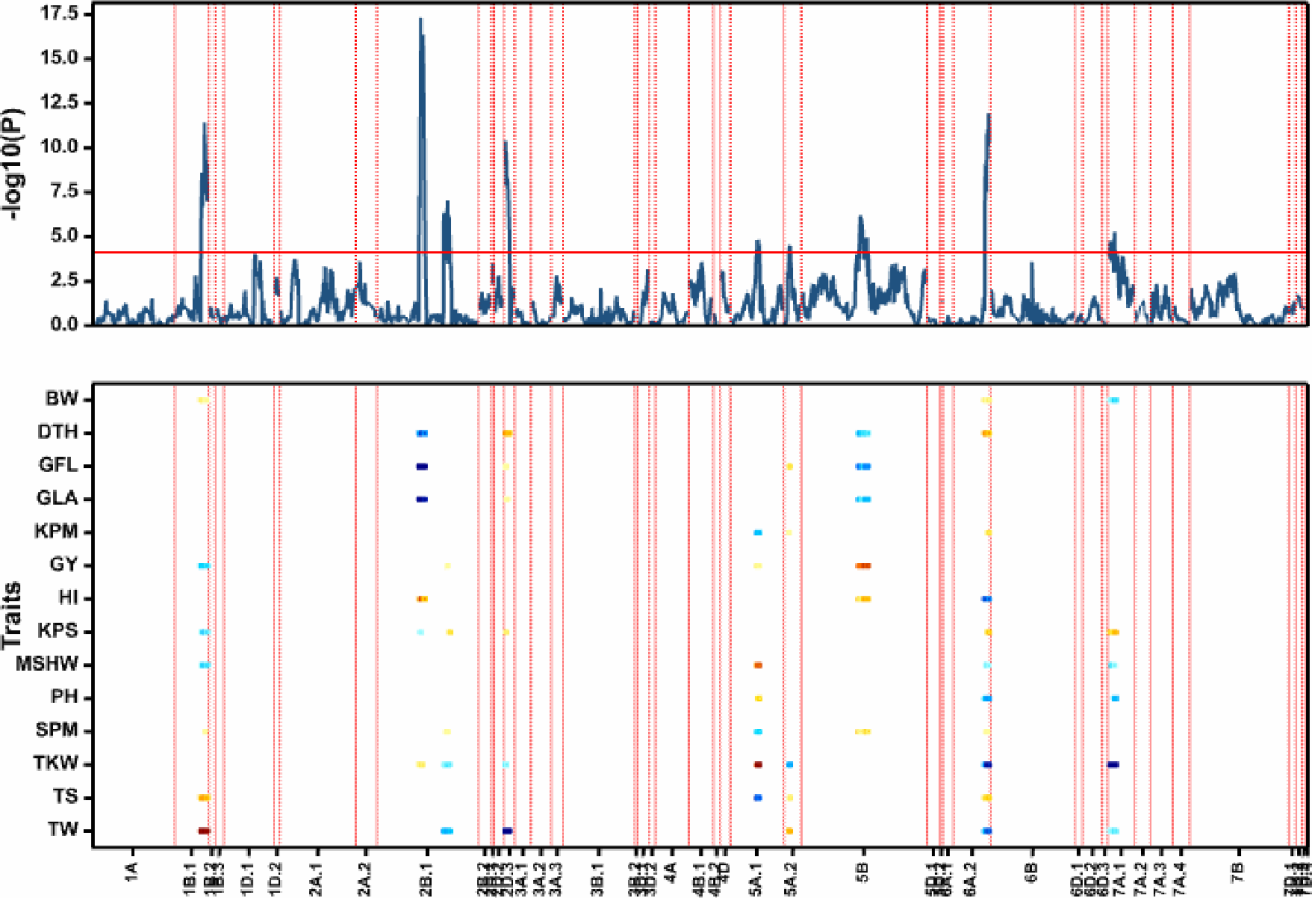
Genomewide scan for multi-trait QTL for grain yield and agronomic traits. The upper graph is the QTL profile plot with the y-axis representing the log of likelihood, -log (P), for declaring significance of QTL. The red horizontal line represents the threshold corrected for the number of independent tests using Li and Ji [38]. The lower profile is the genomewide heat map of significant QTL across environments. The *y*-axis is the traits and the *x*-axis represents the chromosomes. The light blue to blue color indicates the high value allele originates from CO9602932 and the yellow-red color indicates the high value allele originates from TAM 111.

The multi-trait genetic model across seven environments revealed nine significant QTL with all QTL detected showing significant QTI as indicated by a color coded HVA (red color = HVA from TAM 111, blue = HVA from CO960293-2) [10, 12] (Table 2, Fig 2).Seven of the nine multi-trait QTL were also detected based on M1 and M2 approach (Table 1). Multi-trait QTL on chromosomes 2B (*Qmt*.*tamu*.*2B*.*1)* and 6A.2 (*Qmt*.*tamu*.*6A*.*2)* were associated with the highest number of traits suggesting that these genomic regions are essential in wheat breeding for higher yield (Fig 2).

**Table 2:** Multitrait QTL detected using data pooled across environments.

QTL Name^a^	QTL ID	Chr	Position (cM)	Peak SNP	CI_LL^b^	CI_UL^c^	-log10(P)	Traits^d^	AE^e^	PVE^f^ (%)	HVA^g^	Detected in single environment analysis^h^	Detected in multi- environment
*Qmt*.*tamu*.*1B*.*1*	18	1B.1	111.4	IWB37118	91.3	123.1	10.9	BW, GY, KPS, MSHW, TS, TW	0.17, 0.23, 0.18, 0.19, 0.24, 3.60	3.0, 5.4, 3.3, 3.7, 1.1, 8.0	P2, P1, P1, P1, P2, P2	Yes: for TW in HY13 and ET13	No
*Qmt*.*tamu*.*2B*.*1*	5	2B.1	165.5	IWB43273	163.5	167.5	17.3	DTH, GFL, GLA, HI, TKW, TW	0.43, 0.76, 0.63, 0.48, 0.35, 2.15	18.4, 57.7, 39.2, 22.9, 12.0, 2.8	P1, P1, P1, P2, P2, P2	Yes: for GFL and GLA in BS14; HI, GFL, GLA, and GY in CH14; DTH and HI in ET13; DTH, TW, and TKW in HY13	Yes: for DTH, GFL, GLA, GY, HI, and SPM
*Qmt*.*tamu*.*2B*.*1*.*1*	19	2B.1	269.3	IWB70591	221.3	317.3	7.6	GY, SPM, TKW, TW	0.19, 0.15, 0.16, 2.96	3.5, 2.2, 2.4, 5.2	P2, P2, P1, P1	Yes: for GY in ET13	No
*Qmt*.*tamu*.*2D*.*3*	6	2D.3	1.2	IWB26013	0.0	8.4	9.9	DTH, GFL, GLA, TW	0.27, 0.10, 0.11, 0.42	7.5, 1.0, 1.1, 17.7	P2, P2, P2, P1	Yes: for TW in ET13, ET14, and HY13; DTH in AB13	Yes: for TW
*Qmt*.*tamu*.*5A*.*1*	8	5A.1	100.4	IWB912	83.6	117.2	5.1	KPM, GY, MSHW, PH, SPM, TKW, TS	0.19, 0.20, 0.24, 0.15, 0.15, 0.27, 0.30	3.7, 4.0, 5.6, 2.2, 2.1, 7.3, 9.0	P1, P2, P2, P2, P1, P2, P1	Yes: for KPM in BS14	Yes: for GY
*Qmt*.*tamu*.*5A*.*2*	9	5A.2	17.7	IWB6809	0.0	56.4	4.9	GFL, KPM, TKW, TW	0.09, 0.18, 0.20, 2.19	0.8, 3.4, 3.9, 2.8	P2, P2, P1, P2	Yes: for TKW in BS14; KPM in CH14; TW in HY13	Yes: for KPM, KPS, and TKW
*Qmt*.*tamu*.*5B*.*1*	11	5B	226.5	IWB21839	201.2	251.8	7.3	DTH, GFL, GLA, GY, HI, SPM	0.16, 0.15, 0.12, 0.26, 0.14, 0.15	2.7, 2.4, 1.4, 7.0, 2.0, 2.2	P1, P1, P1, P2, P2, P2	Yes: for GY in HY13	Yes: for GY
*Qmt*.*tamu*.*6A*.*2*	14	6A.2	130.7	IWB26244	111.6	133.5	11.8	DTH, HI, KPS, MSHW, PH, SPM, TKW, TS, TW	0.14, 0.23, 0.13, 0.15, 0.25, 0.13, 0.29, 0.17, 3.35	1.9, 5.1, 1.6, 2.2, 6.3, 1.7, 8.3, 2.9, 6.9	P2, P1, P2, P1, P1, P2, P1, P2, P1	Yes: for SPM and TKW in ET13; TKW in ET14; TKW, TW, and PH in HY13	Yes: for HI, PH, and TKW
*Qmt*.*tamu*.*7A*.*1*	16	7A.1	20.6	IWB7632	2.9	38.3	4.9	BW, KPS, PH, TKW	0.17, 0.18, 0.19, 0.30	3.0, 3.3, 3.7, 8.7	P1, P2, P1, P1	Yes: GY in ET14; KPS and TKW in HY13	Yes: for KPS and TKW

^a^ QTL name including trait, institute, and chromosome location; the chromosome location part is unique for each numbered QTL if the peak positions were less than 40 cM; within each chromosome fragment, different numbered QTL will have various chromosome fragment parts starting from the fragment name, then adding “.1, .2, …”.

^b^ CI_LL, lower limit of QTL confidence interval in centiMorgans.

^c^ CI_UL, upper limit of QTL confidence interval in centiMorgans.

^d^ Abbreviation of traits: GY grain yield, TW test weight, DTH days to heading, PH plant height, HI harvest index, KPM kernels m^2^, BW biomass weight, SPM spike m^2^, KPS kernels spike^1^, MSHW mean single head weight, TKW, thousand kernel weight, TS total stems, GLA green leaf area, GFL greenness of flag leaf

^e^ Additive effects corresponding to each trait in the trait column.

^f^ PVE, phenotypic variance explained (%) corresponding to each trait in the trait column.

^g^ HVA, High value allele corresponding to each trait in the trait column. P1 = CO960293-2, P2 = TAM 111.

^h^ ENV, environment, AB13 Aberdeen 2013, BS14 Bushland 2014, CH14 Chillicothe 2014, ET13 Etter 2013, ET14 Etter 2014, HY13 Hays 2013, HY14 Hays 2014.

Grain yield was linked to multi-trait QTL on chromosome 1B.1, 2B.1, 5A.1, and 5B with all QTL except 1B.1 showing HVA from TAM 111 (Fig 2). Biomass weight was linked to multi-trait QTL on chromosome 1B.1 and 7A.1 (Table 2 and Fig 2). Multi-trait QTL associated with plant height were detected on chromosome 5A.1, 6A.2, and 7A.1 and the chromosomal location of multi-trait QTL for plant height on 6A.2 agreed with single trait multi-environment QTL model (Tables 1 and 2). Days to heading was linked to multi-trait QTL on 2B.1, 2D.3, 5B, and 6A.2 whereas green leaf area and greenness of the flag leaf were associated with multi-trait QTL on chromosome 2B.1, 2D.3, and 5B although the greenness of the flag leaf had an additional QTL on 5A.2 (Table 2). The multi-trait QTL with significant additive effect on kernels m^2^ were mapped on chromosome 5A.1 and 5A.2 (Table 2). Harvest index was associated with multi-trait QTL on 2B.1, 5B, and 6A.2 whereas kernel spike^-1^ QTL with significant additive effects were detected on 1B.1, 6A.2, and 7A.1 (Table 2). Mean single head weight was linked to multi-trait QTL on chromosomes 1B.1, 5A.1, and 6A.2. Thousand kernel weight QTL were mapped on chromosome 2B.1, 5A.1, 5A.2, 6A.2, and 7A.1 (Table 2). The QTL for thousand kernel weight detected on 2B.1, 5A.2, 6A.2, and 7A.1 were also detected in a single trait multi-environment model (Table 1). The multi-trait QTL linked to spikes m^2^ were detected on chromosome 2B.1, 5A.1, 5B, and 6A.2 with all QTL showing HVA from TAM 111 except for the QTL on 5A.1. Test weight was associated with multi-trait QTL on 1B.1, 2B.1, 2D.3, 5A.2, 6A.2, and 7A.1 whereas the multi-trait QTL with significant additive effect on total stems were detected on chromosome 1B.1, 5A.1, and 6A.2 (Table 2). The multi-trait QTL on 1B.1, 2B.1, 2D.3, 6A.2, and 7A.1 were associated with kernels spike^1^. However, a test for significance of additive revealed that only the QTL on 1B.1, the multi-trait QTL on 6A.2 and on 7A.1 had significant additive effect on kernels spike^1^. Mean single head weight was associated with multi-trait QTL on 1B.1, 5A.1, 6A.2, and 7A.1 although the latter had nonsignificant additive effect.

The co-location observed in multi-trait analysis was supported by results from previous analysis. In M1 analysis, mean single head weight, spikes m^2^ and harvest index were co-located on chromosome 1A from 173.0 cM to 202.9 cM whereas thousand kernel weight, kernels m^2^, and kernels spike^1^ were co-located on 5A.2 from 1.0 cM and 24.7 cM, respectively based on peak SNP position (S4 Table and S1–S3 Figs). Test weight and kernels spike^1^ were co-located on chromosome 2A.1 at 51.8 cM and 53.7 cM, respectively (S4 Table and S1–S3 Figs). QTL for days to heading, greenness of the flag leaf, green leaf area, grain yield, harvest index, spikes m^2^, thousand kernel weight, and test weight were co-located from 165.5 cM to 172.9 cM on chromosome 2B.1. In addition, thousand kernel weight, plant height, and harvest index were co-located on 6A.2 from 115.3 cM to 129.7 cM (S4 Table and S1–S3 Figs). In M2 QTL model showed colocation at 168–175 cM of 2B.1 for greenness of the flag leaf and green leaf area and in the same map position as M1 colocation (Table 1 and S4 and S5 Tables). Spikes m^-2^ and thousand kernel weight were co-located on chromosome 6A.2 in ET13. In HY13, colocation of QTL was observed for test weight and thousand kernel weight on 2B.1; thousand kernel weight, test weight, and plant height on 6A.2; and kernel spike^-1^ and thousand kernel weight on 7A.1 (S4 and S5 Tables). The colocation of QTL for different traits could partly explain the genetic correlation observed in this study (Fig 1D).

### Combined analysis of pleiotropy and epistasis

**Fig 3:**
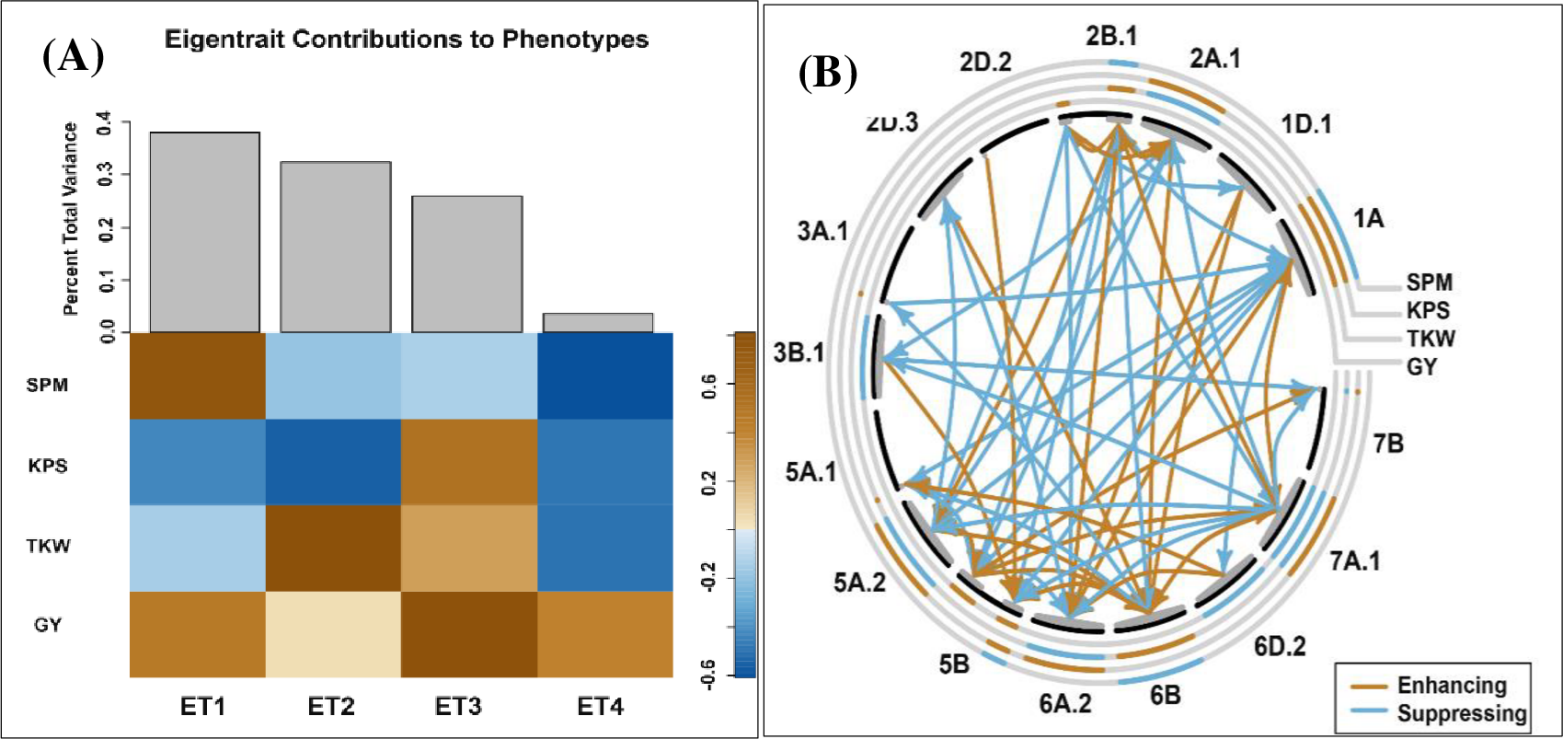
Interactions and pleiotropic patterns based on markers linked to significant QTL from multi-environment and multi-trait QTL analysis. (**A)** Eigentraits (ET) generated through singular value decomposition of phenotypic data. The first three ET explained approximately 90% of the phenotypic variation and were used for combined analysis of pleiotropy and epistasis (CAPE). The legend indicates the direction of variance among traits. For instance, for ET1, kernel spike^-1^ (KPS) and thousand kernel weight (TKW) vary in opposite direction compared to grain yield (GY) and spike m^-2^ (SPM). (**B)** Interaction patterns from CAPE with main effects (brown and blue) depicted on the concentric rings and interactions depicted by color-coded arrow lines within the plot. Brown arrow line represents enhancing effect whereas blue arrow line indicates repressing effect.

Combined analysis of pleiotropy and epistasis (CAPE) jointly uses multiple phenotype and genetic markers to model and define marker-to-trait effects and marker-to-marker interactions. The detected marker-to-trait effect and marker interactions are defined either as enhancing (allele from male parent) or repressing (allele from female parent). Typically, the linear independence of the phenotypic data is achieved by singular value decomposition (SVD) to extract composite traits (eigentraits) prior to analysis [13, 39, 40]. In this study, SVD of grain yield and three yield components (spikes m^-2^, kernels spike^-1^, and thousand kernel weight) generated four eigentraits (ET). The first three ET accounted for approximately 90% of the variation in the phenotype and were used for CAPE to elucidate interaction patterns underlying grain yield and yield components. The black arc lines represent linkage groups. Light grey concentric lines represent traits with the innermost concentric line representing grain yield followed sequentially by yield components namely thousand kernel weight, kernels spike^-1^, and spikes m^-2^ (Fig 3B). A network of marker-to-marker interaction is represented by color-coded arrow line depending on whether the interaction is enhancing (brown) or repressing (blue). The segment of the linkage group involved in the interaction is marked by grey color inside the black arc. The main effect calculated based on a subset of markers (markers associated with significant effect in the previous section) are mapped along the concentric lines. Both positive and negative pleiotropic effects were observed on chromosomes 1A, 2A.1, 2B.1, 5A.2, 5B, 6A.2, 6B, 7A.1, and 7B (Table 3 and Fig 3B). Markers on 2B.1, *IWB70591* and *IWB64246*, had enhancing effect on grain yield. In addition, *IWB23950* at 228.0 cM and *IWB52093* at 402.6 cM on chromosome 5B had enhancing effect on grain yield. On the contrary, *IWA3983* on 3B.1, *IWB31561* on 6D.2, and *IWB11000* on 7A.1 had a repression effect on grain yield (Table 3 and Fig 3B). Thousand kernel weight was enhanced by *IWB46316* and *IWB42357* on 1A; *IWB16370* and *IWB8143* on 2B.1; *IWB47055* on 3A.1; *IWB912* on 5A.1; *IWB72333*, *IWB11040*, *IWB9108* on 6B. QTL that had repression effect on thousand kernel weight include *IWB7015* on 2A.1; *IWB74769* on 5A.2; *IWB7004* on 6A.2; and *IWA7406* and *IWB11000* on 7A.1 (Table 3). The two markers, *IWB46316* and *IWB42357* on 1A, enhancing thousand kernel weight had positive pleiotropic effect on kernels spike^1^ but had repression effect on spikes m^2^. In addition, the *IWB16370* on 2B.1 which had positive effect on thousand kernel weight showed negative pleiotropic effect on spikes m^2^ (Table 3; Fig 3B). Besides *IWB46316* on 1A, other markers that had enhancing effect on kernels spike^1^ include *IWB13287* and *IWB42357* on 1A; *IWB7015* and *IWB28453* on 2A.1; *IWB12338*, *IWB8687* and *IWB14407* on 5A.2; *IWB29746* on 5B; *IWB7004* on 6A.2; *IWB41660* and *IWB7632* on 7A.1 (Table 3 and Fig 3B). *IWB7015*, *IWB7004*, and *IWB10879* enhancing KPS had a negative pleiotropic effect on thousand kernel weight.

**Table 3:** Significant marker-to-trait influences based on a subset of markers in combined analysis of pleiotropy and epitasis.

Markers	QTL ID	Source linkage group	Source position	Target traits^a^	Effect on target	Source of alleles	Effect	SE	|Effect|/SE^b^	P-value	q-value
IWB70591	19	2B.1	269.3	GY	Enhancing	TAM 111	0.59	0.18	3.23	0.0013	0.0392
IWB64246	19	2B.1	289.6	GY	Enhancing	TAM 111	0.66	0.19	3.47	0.0005	0.0270
IWB23950	11	5B	228.0	GY	Enhancing	TAM 111	0.84	0.20	4.23	0.0000	0.0008
IWB52093	12	5B	402.6	GY	Enhancing	TAM 111	0.96	0.32	3.02	0.0025	0.0360
IWB46316	21	1A	148.1	TKW	Enhancing	TAM 111	0.63	0.19	3.27	0.0013	0.0440
IWB42357	1	1A	196.0	TKW	Enhancing	TAM 111	0.78	0.19	4.08	0.0001	0.0015
IWB16370	4	2B.1	122.7	TKW	Enhancing	TAM 111	0.79	0.20	3.86	0.0002	0.0045
IWB8143	5	*2B*.*1*	*166*.*7*	TKW	Enhancing	TAM 111	0.81	0.20	4.10	0.0000	0.0022
IWB47055	25	*3A*.*1*	*44*.*0*	TKW	Enhancing	TAM 111	0.85	0.21	4.05	0.0001	0.0031
IWB912	8	5A.1	97.1	TKW	Enhancing	TAM 111	0.67	0.19	3.52	0.0006	0.0104
IWB72333	15	6B	127.5	TKW	Enhancing	TAM 111	0.79	0.21	3.76	0.0002	0.0061
IWB11040	15	6B	145.7	TKW	Enhancing	TAM 111	0.78	0.21	3.67	0.0003	0.0068
IWB9108	26	6B	230.7	TKW	Enhancing	TAM 111	0.67	0.18	3.71	0.0003	0.0081
IWB46316	21	1A	148.1	KPS	Enhancing	TAM 111	0.58	0.18	3.18	0.0017	0.0298
IWB13287	1	1A	169.9	KPS	Enhancing	TAM 111	0.76	0.21	3.57	0.0005	0.0096
IWB42357	1	1A	196.0	KPS	Enhancing	TAM 111	0.70	0.20	3.52	0.0005	0.0136
IWB7015	3	2A.1	43.9	KPS	Enhancing	TAM 111	0.97	0.21	4.51	0.0000	0.0001
IWB28453	3	2A.1	67.1	KPS	Enhancing	TAM 111	0.65	0.19	3.47	0.0006	0.0073
IWB12338	9	5A.2	1.0	KPS	Enhancing	TAM 111	0.61	0.20	3.14	0.0019	0.0243
IWB8687	9	5A.2	13.2	KPS	Enhancing	TAM 111	0.73	0.20	3.57	0.0005	0.0215
IWB14407	9	5A.2	24.7	KPS	Enhancing	TAM 111	0.70	0.20	3.44	0.0007	0.0342
IWB29746	12	5B	384.8	KPS	Enhancing	TAM 111	0.67	0.19	3.52	0.0005	0.0165
IWB7004	14	6A.2	128.7	KPS	Enhancing	TAM 111	0.63	0.19	3.40	0.0008	0.0312
IWB41660	16	7A.1	11.2	KPS	Enhancing	TAM 111	0.60	0.18	3.40	0.0008	0.0165
IWB7632	16	7A.1	20.6	KPS	Enhancing	TAM 111	0.71	0.21	3.34	0.0010	0.0190
IWB10879	17	7B	12.7	KPS	Enhancing	TAM 111	0.59	0.19	3.06	0.0025	0.0443
IWA3983	7	3B.1	107.9	GY	Repressing	CO960293-2	-0.69	0.19	3.52	0.0005	0.0164
IWB31561	27	6D.2	41.1	GY	Repressing	CO960293-2	-0.69	0.19	3.55	0.0004	0.0217
IWB11000	43	7A.1	57.8	GY	Repressing	CO960293-2	-0.61	0.20	3.11	0.0019	0.0298
IWB7015	3	2A.1	43.9	TKW	Repressing	CO960293-2	-0.71	0.20	3.58	0.0005	0.0108
IWB74769	9	5A.2	24.3	TKW	Repressing	CO960293-2	-0.93	0.18	5.10	0.0000	0.0000
IWB7004	14	6A.2	128.7	TKW	Repressing	CO960293-2	-0.97	0.19	5.08	0.0000	0.0000
IWA7406	16	7A.1	5.7	TKW	Repressing	CO960293-2	-0.74	0.17	4.31	0.0000	0.0008
IWB11000	43	7A.1	57.8	TKW	Repressing	CO960293-2	-0.66	0.17	3.87	0.0002	0.0055
IWB10879	17	7B	12.7	TKW	Repressing	CO960293-2	-0.67	0.17	3.88	0.0001	0.0034
IWB46316	21	1A	148.1	SPM	Repressing	CO960293-2	-0.96	0.21	4.47	0.0000	0.0006
IWB13287	1	1A	169.9	SPM	Repressing	CO960293-2	-0.74	0.18	4.04	0.0001	0.0033
IWB42357	1	1A	196.0	SPM	Repressing	CO960293-2	-0.87	0.19	4.59	0.0000	0.0003
IWB16370	4	2B.1	122.7	SPM	Repressing	CO960293-2	-0.69	0.20	3.46	0.0007	0.0343
IWA4416	12	5B	478.6	SPM	Repressing	CO960293-2	-0.59	0.17	3.35	0.0010	0.0420
IWB72333	15	6B	127.5	SPM	Repressing	CO960293-2	-0.76	0.19	4.05	0.0001	0.0016
IWB8809	15	6B	166.4	SPM	Repressing	CO960293-2	-0.70	0.18	3.83	0.0002	0.0025
IWB9108	26	6B	230.7	SPM	Repressing	CO960293-2	-0.82	0.18	4.63	0.0000	0.0002

^a^ Abbreviation for traits: GY grain yield, TKW thousand kernel weight, KPS Kernels spike^1^, SPM Spikes m^2^

^b^ The test statistic was computed by dividing the absolute effect by standard error (SE). The likelihood test for chance association was calculated using 500,000 permutations. The test statistic was compared with null distribution generated through permutation. To minimize inflation of false positives due to multiple tests, the false discovery rate (FDR) corrected P-values (q-values) were computed

Marker-to-marker interaction patterns were depicted using color-coded arrowed lines with direction of the arrow indicating the target markers enhanced or repressed (Fig 3B). Similar to marker-to-trait effect, the brown arrow lines indicate favorable interactions (enhancing) whereas blue lines indicate unfavorable interactions (suppressing). The effect of markers on 1A was enhanced by interaction with markers on 7A.1. The marker *IWB7015* on 2A.1 was enhanced by interaction with *IWB7053* and *IWB64246* on 2B.1. Markers on 2B.1 were enhanced by interaction with markers on 2A.1, 2B.1, 6A.2, and 7A.1. *IWB912* on 5A.1 was enhanced by *IWB6263* whereas a set of markers mapped from 1.0 to 17.7 cM on 5A.2 were enhanced by interacting with several markers on 1A, 2B.1, 5A.1, and 5B (Fig 3B and S7 Table). On chromosome 5B, *IWB21839*, *IWB59433*, *IWB29746*, and *IWB52093* were enhanced by several markers on 5A.2, 6B, 2B.1, 2D.2, and 3B.1 (Fig 3B and S7 Table). Several markers on 6A.2 were enhanced by interaction with markers on 1A, 1D.1, 2B.1, and 6D.2. In addition, *IWB43368* mapped at 112.7 cM on 6A.2 had enhancing effect on *IWB1295* mapped at 129.7 cM on the same chromosome. A number of markers detected on 1D.1, 2A.1, 2D.3, 5A.2, 5B, and 7A.1 enhanced markers detected on 6B. *IWB7632* on 7A.1 was enhanced by *IWB46316*, *IWB13287*, and *IWB42357* on 1A. *IWB41660* showed positive interaction with *IWB65641* on 5B whereas *IWA7406* on 7A.1 showed positive interaction with *IWB72333* and *IWB8809* on *6B*. Marker *IWB65641* on 5B enhanced *IWB10879* on 7B (Fig 3B and S7 Table). Negative marker-to-marker interactions with sources of alleles from CO9602932 were detected between 10 source chromosomes including 1A, 2A, 2B, 3A, 3B, 5A, 5B, 6A, 6B, and 7A (Fig 3B and S7 Table).

## Discussion

We applied different genetic models to define the underlying genetic basis of quantitative traits in winter wheat evaluated in different geographical testing footprints in the US Great Plains. A single trait multi-environment QTL analysis revealed that most traits had significant QEI, underscoring the need of multi-environment phenotyping to account for this variation. Across all the models and traits, we detected 43 unique QTL with most QTL showing colocation or pleiotropic effect. Nine of these QTL were consistent across environment and QTL analysis methods and seven of the nine QTL were also detected based on multi-trait QTL analysis approach. Stable grain yield QTL were detected on chromosomes 2B (*Qgy*.*tamu*.*2B*.*1*) and 5B (*Qgy*.*tamu*.*5B*.*1*) based on M1, M2, M3, and M4. The multi-trait QTL on 2B.1 was mapped at position 165.5 cM (172.9 cM in M1 analysis) and had a significant effect on grain yield and other traits (Tables 1 and 2, S2–S7 Tables, S1–S3 Figs). Based on genetic map position, *Qgy*.*tamu*.*2B*.*1* was either pleiotropic or co-located with QTL for days to heading, test weight, kernel weight, spikes m^2^, greenness of the flag leaf, green leaf area, and harvest index. BLAST search using peak marker for *Qgy*.*tamu*.*2B*.*1* revealed that it is associated with several genes including NAD-dependent histone deacetylase domain containing protein and heat shock 70 kDa protein (S8 Table). Four other multi-trait QTL (*Qmt*.*tamu*.*1B*.*1*, *Qmt*.*tamu*.*2B*.*1*.*1*, *Qmt*.*tamu*.*5A*.*1*, and *Qmt*.*tamu*.*5B*.*1*) were also linked to grain yield and other traits with *Qmt*.*tamu*.*5B*.*1* showing map position consistency with QTL analysis in M1. Comparison of genetic maps in Wu et al. [41] and the present study, both constructed using 90K SNP, showed that IWA4518 (*BobWhite_c8113_532*) was mapped at 219.6 cM in the present work and falls within the confidence interval for the multi-trait QTL *Qmt*.*tamu*.*2B*.*1*.*1*. The base sequence for IWA4518 is within 1181 bp mRNA for uncharacterized protein in *Aegilops tauschii* subsp. *tauschii* and 1264 bp mRNA from *Triticum aestivum* cDNA clone *WT006_C07* (www.ncbi.nlm.nih.gov). This region has also been associated with starch gelatinization in a study using 90K and 660K SNP array [42]. In their study, the linked SNP to the QTL for starch gelatinization was RAC875_c56101_368, which was mapped near *Qmt*.*tamu*.*2B*.*1*.*1* in the present study.

We detected other stable QTL for thousand kernel weight on 6A.2, 6B, and 7A.1. A recent study using 90K SNP array reported kernel weight QTL on 3D, 4A, 5A, 6A, 7A, and 7B based on multi-environment data from Gao [43]. The map position of QTL on 6A, and 7A based on interval SNP were different from the present study suggesting that these could be new QTL for thousand kernel weight. A consistent QTL for mean single head weight (*Qmshw*.*tamu*.*1A*) was detected on 1A explaining 4.1–10.1% of the phenotypic variation. This region was linked to spike compactness and sterile spikelet number in a RIL population genotyped using 90K SNP array and SSR markers [44]. Plant height was linked to two stable QTL, one at 142 cM on 2B.1 and one on 6A.2 clustered or had pleiotropic effect on kernel weight, green leaf area, harvest index, and spikes m^2^. Recent work by Wurschum et al [2] and Tian et al [45] showed that chromosome 6A harbors *Rht24* gene for reduced plant and that the gene is sensitive to gibberellic acid. These studies used SNP, GBS and SSR making it difficult to make direct comparison with the stable QTL for plant height detected in the present work although both QTL were mapped on the long arm of chromosome 6A. A summary of QTL by Triticeae Coordinated Agricultural Project (TCAP) reported. that *IWB6528* (*BS00012081_51*), *IWB56597* (*RAC875_c31358_214*), *IWB4233* (*BobWhite_c6771_697*), and *IWB54163* (*RAC875_c15844_348*) were associated with PH QTL on chromosome 2B (https://triticeaetoolbox.org/wheat/qtl/qtl_report.php). In the present work, the four SNP were mapped within the confidence interval (119.4–165 cM) for plant height QTL on chromosome 2B suggesting that this could be same QTL for plant height. The peak region for *2B*.*1*.*2* (IWB62653) was mapped at 142.2 cM compared to 144.6 cM, 144.7 cM, 144.0 cM and 145.1 for *IWB6528*, *WB56597*, *IWB4233*, and *IWB54163*, respectively. Megablast search of the IWB62653 sequence revealed that it is within 1656 bp mRNA of *wre1n*.*pk0115*.*e3*:*fis* clone (www.ncbi.nlm.nih.gov).

We detected stable QTL for test weight on chromosome 2A, 2B.1 at 164 cM and 6A.2. Both test weight QTL on 2B and 6A were also detected when data was analyzed using multi-trait QTL model with additional QTL *Qmt*.*tamu*.*1B*.*1* on 1B, *Qmt*.*tamu*.*2D* on chromosome 2D, and *Qmt*.*tamu*.*5A*.*1* on chromosome 5A (Table 3). In a previous study, IWB47726 *(Kukri_c7770_176*) and IWB73106 (*Tdurum_contig68343_339*) were associated with fertile spikelet number and spikelet compactness, respectively [44]. These two SNPs were mapped within the confidence interval for mult-trait QTL *Qmt*.*tamu*.*1B*.*1* in the present study.

The significant QTI observed in multi-trait model suggests that pleiotropy and epistatic interactions can contribute to significant variations. Empirical studies on epistasis have reported varying results with some reporting significant contribution of epistasis in the modulation of quantitative traits while other studies have shown nonsignificant contribution [46]. Results of the multi-trait model were supported by further genetic analysis based on combined analysis of pleiotropy and epistasis which showed various patterns of genetic interactions among markers and traits. The marker-to-trait effect revealed that grain yield was enhanced by markers on 2B.1 and 5B with favorable alleles from TAM 111 but was repressed by a set of markers detected on 3B.1, 6D.2, and 7A.1 with alleles from CO9602932. Two markers, IWB46316 and IWB42357 on 1A enhancing thousand kernel weight had positive pleiotropic effect on kernels spike^-1^ but had negative pleiotropic effects on spikes m^2^ (Fig 3B and Table 3). Markers enhancing kernels spike^-1^ on 2A.1, 6A.2, and 7A.1 had a negative pleiotropic effect on thousand kernel weight. These results suggest the importance of epistasis and pleiotropy in the genetic architecture of yield components in winter wheat.

QTLNetwork analyses confirmed some results of epistasis from CAPE analyses and provided information on the importance QTL by environment and epistasis by environment interactions. A × A, A × E, and A × A × E interactions were identified for several traits across environments in this study. These interactions could be helpful in evaluation of the major QTL and potential candidate genes in marker-assisted breeding and genomic prediction. Validation and conversion to KASP of SNPs tightly linked to yield and its components will be useful for their utility in marker-assisted breeding.

## Conclusions

We have provided QTL discovery results using both multi-environment, individual environment, and multi-trait analysis models of GenStat as well as using MapQTL and QTLNetwork. The multi-environment QTL for grain yield on 5B was repeatable in six out of eight environments. This could be an important target region for fine mapping and validation to identify SNPs associated with grain yield in wheat. A potential target region linked to mean single head weight was detected on 1A. The QTL for mean single head weight on 1A was constitutively expressed in all environments where data was recorded. Several QTL associated with multiple traits were detected through multi-trait QTL analysis approach. Multi-trait QTL on 2B.1 and 6A.2 were associated with the highest number of traits suggesting essential function of these genomic regions. The colocation observed in multi-environment QTL analysis and the results of multi-trait agreed in part with the genetic correlation observed in this study. Beyond QTL discovery, we defined both positive and negative marker-to-marker and marker-to-trait influences detected through joint analysis of pleiotropy and epistasis. Grain yield was enhanced by markers on chromosome 2B.1 and 5B with favorable alleles from TAM 111 but was repressed by several markers on 3B.1, 6D.2, and 7A.1 with favorable alleles from CO9602932. Other traits showed similar patterns of interactions. The results from combined analysis of pleiotropy and epistasis together with the standard QTL analysis provides a platform to build a set of QTL with enhancing effects on the trait of interest. The information could be useful in identification of markers for validation and subsequent deployment to improve grain yield as well as other traits in wheat.

## Supporting information

S1 Fig Genomewide QTL scan for single trait across the multiple environments. (DOCX)

S2 Fig Genomewide QTL scan for single trait across the multiple environments for yield components. (DOCX)

S3 Fig Chromosome fragments significantly linked with quantitative traits with their intervals identified from single trait from multi-environmental model of GenStat. (DOCX)

S1 Table Mean squares, heritability (h2) and mean performance across drought and well-watered environments. (XLSX)

S2 Table Significant additive effects and additive by environment interactions detected using QTLNetwork. (XLSX)

S3 Table Significant additive by additive and epistasis by environment effects of yield and yield components for recombinant inbred lines of CO960293-2/TAM 111 based on data from five environments using QTLNetwork. (XLSX)

S4 Table QTL detected using single trait multi-environment QTL mapping model. Only QTL with significant (P < 0.05) additive effect are shown.
(XLSX)

S5 Table Individual environment quantitative trait loci from GenStat. Only QTL with significant additive effect are shown.
(XLSX)

S6 Table Significant quantitative trait loci of yield and yield components under single environment identified using MapQTL. (XLSX)

S7 Table Marker to marker interactions detected using combined analysis of pleiotropy and epistasis. (XLSX)

S8 Table SNPs from the four consistent unique QTL, their consensus genetic and physical maps, as well as comparative analyses and predicted gene functions. (XLSX)
