# Supplementary material for: Mapping of quantitative trait loci for grain yield and its components in a US popular winter wheat TAM 111 using 90K SNPs
Source: PLoS One. 2017 Dec 21;12(12):e0189669. doi: 10.1371/journal.pone.0189669 (PMC5739412; doi:10.1371/journal.pone.0189669)
Supplement: S2 Fig — (DOCX) [file pone.0189669.s002.docx]

**Fig. S2** Genomewide QTL scan for single trait across the multiple environments for yield components. The upper graph is the QTL profile plot with the y-axis representing the log of likelihood, -log (P), for declaring significance of QTL. The red horizontal represents the threshold corrected for the number of independent tests using Li and Ji (2005). The lower plot is the genomewide heat map of significant QTL across environments. The y-axis is the environments and the x-axis represents the linkage groups. Two vertical dotted lines or a dotted and continuous line delineate a linkage group. The light blue to blue color indicates the high value allele (HVA) originates from CO960293-2 and the yellow-red color indicates the HVA originates from TAM 111. The intensity of the color for QTL corresponds to the magnitude of additive effects with high intensity representing high additive effects. (a) Thousand kernel weight (b) Mean single head weight (c) Spike metre^-2^ (d) Kernels spike^-1^ (e) Greenness of the flag leaf (f) Green leaf area (g) Kernels metre^-2^ (h) Harvest index. Abbreviation of environments: *AB13* Aberdeen 2013, *BS14* Bushland 2014, *CH14* Chillicothe 2014, *ET13* Etter 2013, *ET14* Etter 2014, *HY13* *Hays 2013*, *HY14* Hays 2014, *WA14* Walsh 2014


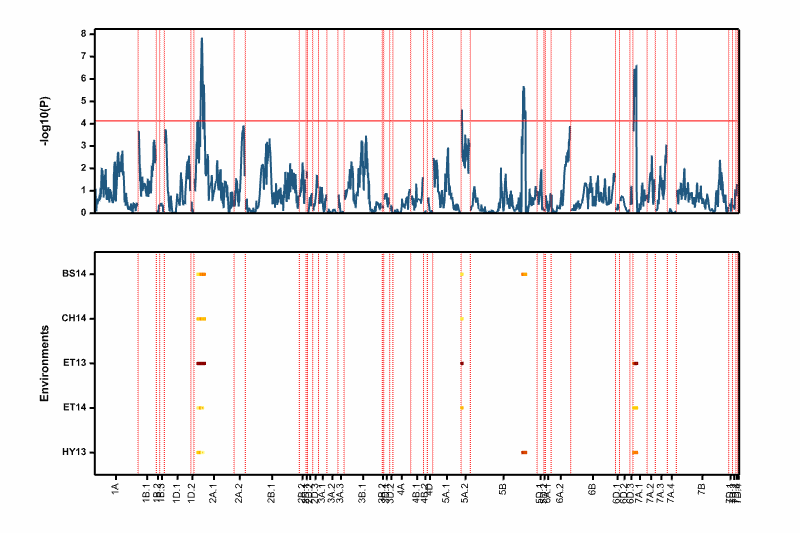
**
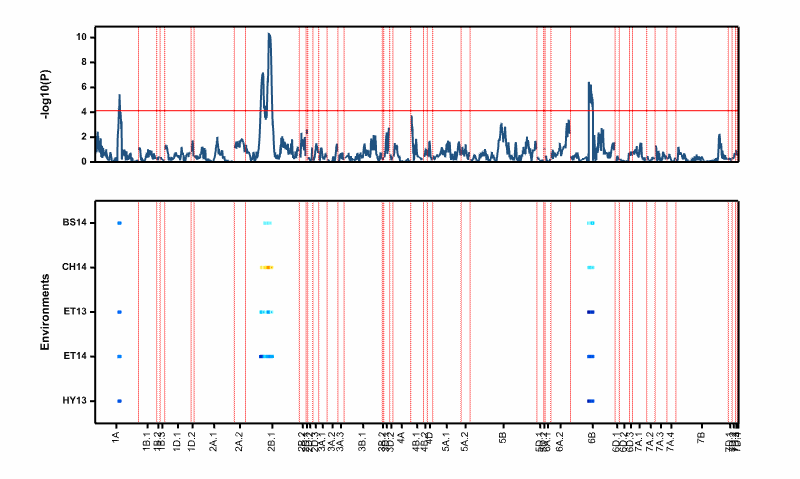
**
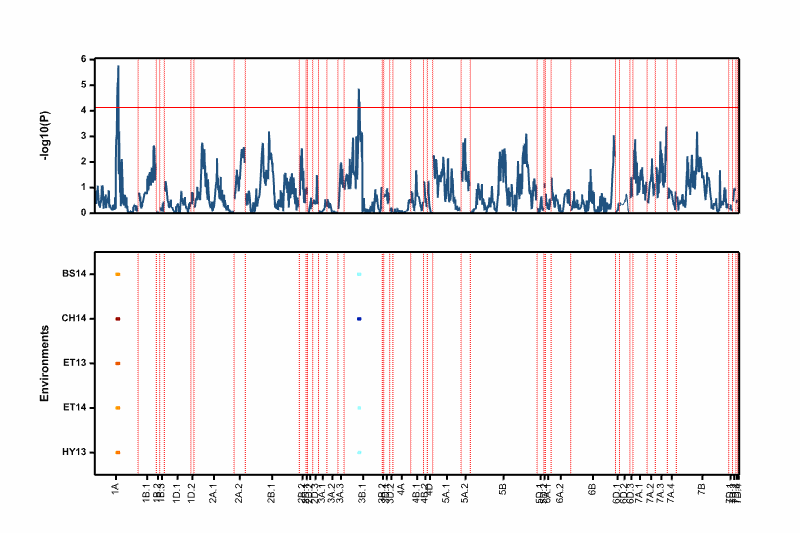

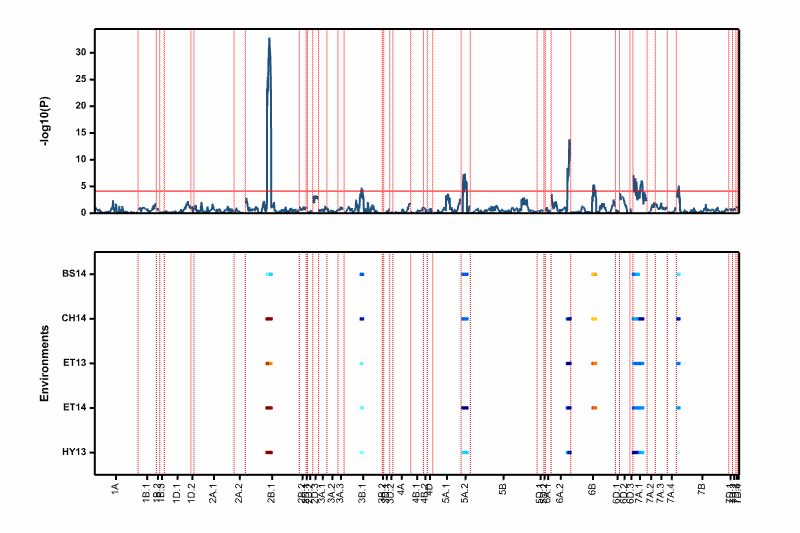


**(d)**

**(c)**

**(b)**

**(a)**


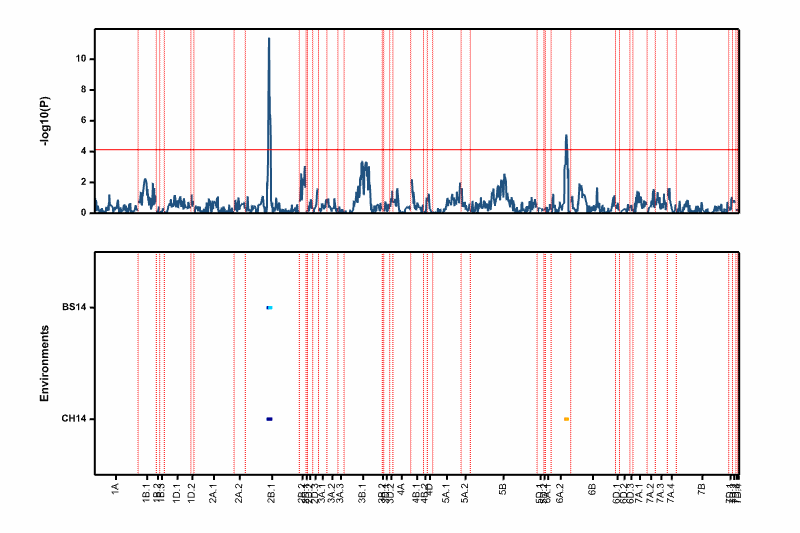
**
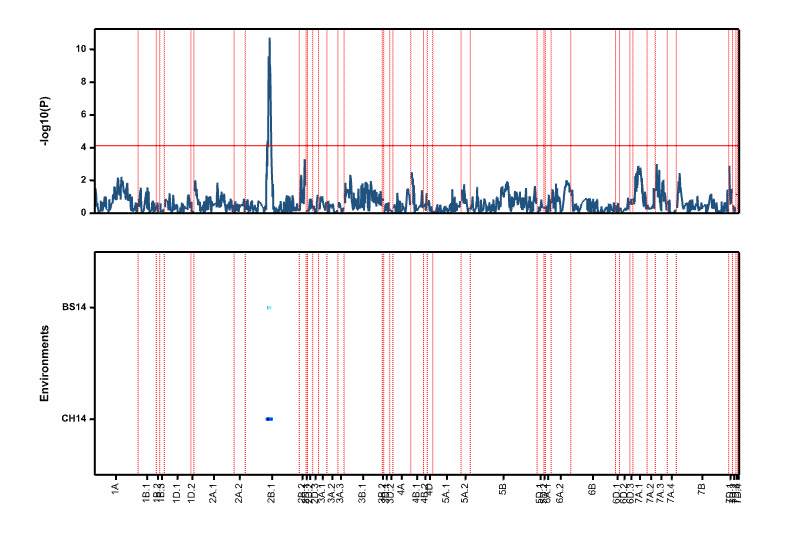
**

**(f)**

**(e)**


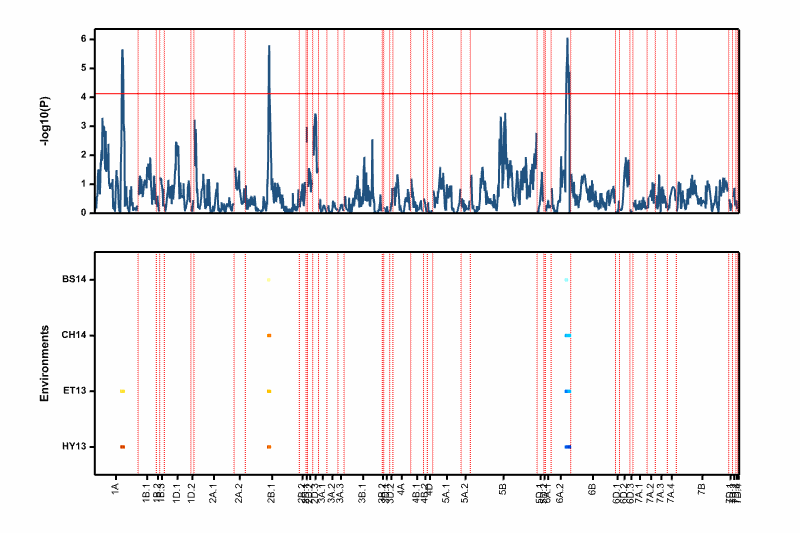

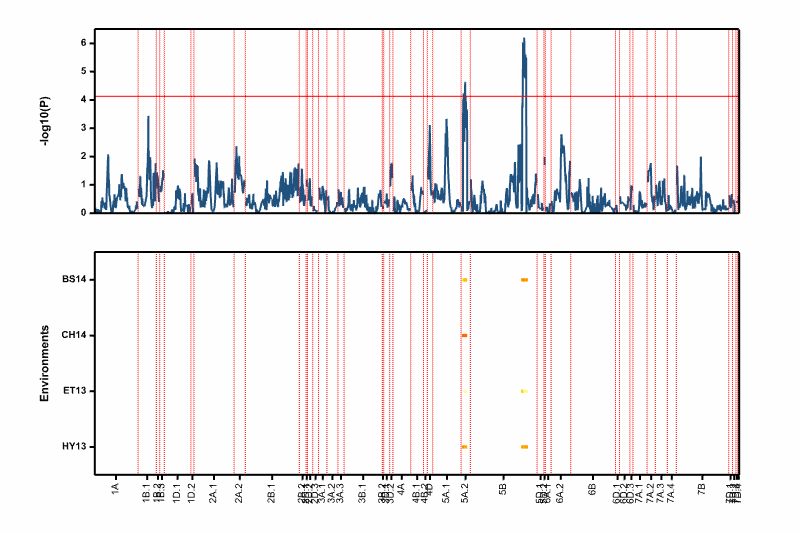


**(g)**

**(h)**
