# Supplementary material for: Mapping of quantitative trait loci for grain yield and its components in a US popular winter wheat TAM 111 using 90K SNPs
Source: PLoS One. 2017 Dec 21;12(12):e0189669. doi: 10.1371/journal.pone.0189669 (PMC5739412; doi:10.1371/journal.pone.0189669)
Supplement: S3 Fig — (DOCX) [file pone.0189669.s003.docx]

S3 Fig. Chromosome fragments significantly linked with quantitative traits with their intervals identified from single trait from multi-environmental model of GenStat.

Abbreviation of traits: *GY* grain yield, *TW* test weight, *DTH* days to heading, *PH* plant height, *HI* harvest index, *KPM* kernels m^-2^, *BW* biomass weight, *SPM* spike m^-2^, *KPS* kernels spike^-1^, *MSHW* mean single head weight, *TKW* thousand kernel weight, *TS* total stems, *GLA* green leaf area, *GFL* greenness of flag leaf

The actual length in cM per chromosome fragment was divided by 4 in order to draw the whole fragment in MapChart (Voorrips, 2002). The QTL were detected based on the multi-env model from GenStat version 17 (15)
